# Cellular Morphology-Mediated Proliferation and Drug Sensitivity of Breast Cancer Cells

**DOI:** 10.3390/jfb8020018

**Published:** 2017-06-06

**Authors:** Ryota Domura, Rie Sasaki, Yuma Ishikawa, Masami Okamoto

**Affiliations:** Advanced Polymeric Nanostructured Materials Engineering, Graduate School of Engineering, Toyota Technological Institute, 2-12-1 Hisakata, Tempaku, Nagoya 468 8511, Japan; ryota11421@gmail.com (R.D.); sd16413@toyota-ti.ac.jp (R.S.); sd13007@toyota-ti.ac.jp (Y.I.)

**Keywords:** spreading parameter, cellular proliferation, drug sensitivity, substrates, cancer cells

## Abstract

The interpretation of the local microenvironment of the extracellular matrix for malignant tumor cells is in intimate relation with metastatic spread of cancer cells involving the associated issues of cellular proliferation and drug responsiveness. This study was aimed to assess the combination of both surface topographies (fiber alignments) and different stiffness of the polymeric substrates (poly(l-lactic acid) and poly(ε-caprolactone), PLLA and PCL, respectively) as well as collagen substrates (coat and gel) to elucidate the effect of the cellular morphology on cellular proliferation and drug sensitivities of two different types of breast cancer cells (MDA-MB-231 and MCF-7). The morphological spreading parameter (nucleus/cytoplasm area ratio) induced by the anthropogenic substrates has correlated intimately with the cellular proliferation and the drug sensitivity the half maximal inhibitory concentration (IC_50_) of cancer cells. This study demonstrated the promising results of the parameter for the evaluation of cancer cell malignancy.

## 1. Introduction

In general, when traction forces are generated in the cells underlying the extracellular matrix (ECM) including artificial substrates, the cells feel the stiffness of the surrounding microenvironment and respond to applied forces, and exert forces in the matrix, in which the traction forces can change cellular morphology and cytoskeletal structure [1].

Understanding the interaction between microenvironment and cancer cells is a critical subject for tackling the metastatic spread of cancer cells and its many associated issues [2,3,4,5,6]. Cancers destroy the normal balance of the microenvironment, which involves the induction of aberrant ECM reconstruction, proliferation, gene expression and migration to promote cancer malignancy [4]. Cancer cells receive mechanical signals (cues) from the aberrant ECM due to the traction force generation, which influences cell fate [7,8]. For this reason, in vitro studies using conventional plastic tissue culture plates are difficult for interpreting the local microenvironment situation. Thus, a new platform (or cancer model) to predict cancer metastatic potential, including cellular proliferation and drug sensitivity, is required to explore a promising cancer therapy. In this regard, the effects of different levels of stiffness of substrates on drug sensitivities of the cancer cells were detected [9,10,11,12,13,14].

Fischbach et al. and Bray et al. reported that the enhancement of tumor vascularization with fewer drug sensitivities was observed when the cancer cells were cultured on porous poly(lactide-co-glycolide) (PLG) scaffolds or glycosaminoglycan based hydrogel [9,10].

Zustiak et al. developed a multiwell polyacrylamide gel-based assay and performed drug screening (paclitaxel) on several cell types. They reported that proliferation and cell spreading area are increased with stiffness, and substrate stiffness affects the cellular response to paclitaxel in some cell types such as HeLa and breast cancer cells (MCF-7) [11]. Schrader et al. reported that hepatocellular carcinoma is less sensitive to cisplatin when they were cultured on softer polyacrylamide gel substrate. Furthermore, a stiffer substrate increases proliferation of hepatocellular carcinoma cells [12]. Nguyen et al. developed the poly(ethylene glycol)-phosphorylcholine hydrogel system and performed a drug resistance test by evaluating the half maximal inhibitory concentration (IC_50_) value of sorafenitib. Drug sensitivity of sorafenib was increased with increasing stiffness and collagen composition of substrate to breast cancer cells (MDA-MB-231) cells [13]. Shin et al. reported that proliferation of human myeloid leukemia cells (K-562) were increased in softer hydrogel when the K-562 encapsulated cells were incorporated into a mouse. Furthermore, drug sensitivity of cytarabine (Ara-C) was also increased for softer hydrogel [14].

According to these previous reports, the stiff substrates tend to increase drug sensitivities of the cancer cells. However, the single factor regarding stiffening substrate is oversimplifying the local microenvironment situation and has not proven to be effective in drug sensitivities for cancer cells.

Herein, we aim to assess the morphological changes of breast cancer cells induced by cell culture substrates with different stiffness and topographies. The combination of both factors of the substrate was employed to mimic local microenvironment situation. Owing to the traction force generation and transmitted stresses to nucleus, the cellular morphologies, such as areas ratio of nuclear and cytoplasm are altered remarkably into non-circular shape. Systematically morphological changes of cancer cells correlated with cellular proliferation, and drug responsiveness were demonstrated.

## 2. Materials, Methods and Results

### 2.1. Materials

A commercial poly(l-lactic acid) (PLLA) with a D content of 0.8% (Mw = 102 kDa, Mw/Mn = 2.71 [15]) kindly supplied by Nagoya municipal industrial research institute was dried under vacuum at room temperature (or 60 °C). A commercial poly(*ε*-caprolactone) (PCL) (Mw = 80 kDa, Mw/Mn < 2) was purchased from Sigma-Aldrich (Tokyo, Japan). Anti-cancer drug of cis-diaminedichloro-platinum (II) (cisplatin) [16] was purchased from Tokyo Chemical Ind. Ltd. (Tokyo, Japan), and *N*-hydroxy-*N′*-phenyl-octanediamide (vorinostat) [17] and erlotinib [18] were purchased from Sigma-Aldrich. All other reagents (dichloromethane (DCM), dimethylformaide (DMF), chloroform (CF), methanol and 1,4-dioxane (DX)) were purchased from Nacalai Tesque (Kyoto, Japan). Millipore Milli Q ultrapure water (specific resistance: 18 MΩcm, total organic carbon (TOC) < 20 ppb, Merck Millipore Japan Co., Tokyo, Japan) through dialysis membrane was used in all experiment.

### 2.2. Preparation of Cell Culture Substrate

#### 2.2.1. Collagen Gel and Collagen-Coated Substrates

For collagen gel substrate, collagen solutions were prepared by mixing solution of 8:1:1 Cellmatrix I-A (Nitta Gelatin Inc., Osaka, Japan)/Minimum essential medium (MEM) (10X) (Life Technologies, Tokyo, Japan) without NaHCO_3_/sterile reconstitution buffer (22 mg/mL of NaHCO_3_, 0.005 M of NaOH, and 200 mM of HEPES (2-[4-(2-hydroxyethyl)piperazin-1-yl]ethane-sulfonic acid; Gibco, Life Technologies), yielding a homogeneous solution at 0 °C. Then, the 30 µL of collagen solution was added onto 96-well plates and heated at 37 °C for 30 min. Collagen-coated substrate were prepared by adding Cellmatrix I-C (Nitta Gelatin Inc.) solution onto 96-well plates and heated at 37 °C for 2 h. Then added solution was removed.

#### 2.2.2. Electrospinning

Both PLLA and PCL nanofibers were fabricated by using electrospinning technique. For PLLA nanofibers, the polymer solution was prepared by dissolving PLLA in DCM and subsequently into DMF (7:3 ratio of DCM/DMF) to obtained a 10 wt. %wt. %wt. % polymer solution. For the preparation of aligned nanofibers, the electrospinning was carried out using a 19-gauge blunt needle (Sansyo Co., Ltd., Tokyo, Japan) mounted on a digital syringe pomp (SPS-2, AS ONE Co., Osaka, Japan) at 16 kV (HST-30K033P-100, Izumi Electric Co., Ltd., Gunma, Japan) and a flow rate of 3.0 mL/h. The rotating collector covered with aluminum foil was used and maintained at a constant distance of 10 cm from the needle. Random nanofibers were collected using a grounded collector of aluminum sheet (15 × 15 cm^2^) at a constant distance of 10 cm from the needle under an operated flow rate of 2.0 mL/h.

For electrospun PCL nanofibers, PCL pellets were dissolved in CF and subsequently into methanol (5:5 ratio of CF/methanol) to obtain an 8.1 wt. %wt. %wt. % polymer solution. Applying voltage and distance from the needle tip were same condition of PLLA nanofiber fabrication. Feeding rates of PCL solution were 2.0 mL/h for aligned fibers and 1.0 mL/h for random fibers, respectively.

Both aligned and random nanofibers were placed on the slide glass of 8-well chamber slide (Watson Co., Ltd., Tokyo, Japan). All substrates were dried overnight in vacuum at room temperature to remove the residual solvents. Then they were sterilized with germicidal UV light for 30 min and further sterilized with 30% ethanol. Finally, all substrates were coated by 2% gelatin (Sigma-Aldrich) to enhance cell adhesion.

#### 2.2.3. Spin Coating

In this study, spin coated substrates were used as a control of electrospun fiber substrates. 4% *w*/*v* PLLA and/or PCL polymer solutions were prepared by dissolving in DX and subsequently spin coated directly (SPINCOATER 1H-D7, MIKASA Co., Ltd., Tokyo, Japan) on slide glass at the rotating speed of 1500 rpm for 15 s. Spin coated substrates were sterilized and coated by using same procedures as described the preparation of the fiber substrate.

### 2.3. Characterization

#### 2.3.1. Fiber Diameter and Orientation

The morphology of PLLA and PCL fiber substrates were observed through a field emission scanning electron microscope (FE-SEM) (SU6600, Hitachi Ltd., Tokyo, Japan). The operated accelerating voltage was 15 kV and the specimens were coated with a thin layer of gold with a thickness of ~20 nm. Both fiber diameter and orientation were analyzed by ImageJ software [19]. Average fiber diameter of each substrate was calculated by measuring 50 individual fibers. To characterize fiber orientation Fast Fourier Transform (FFT) was conducted using ImageJ software by analyzing the FE-SEM images and radial summation of pixel intensities for each angle between 0 and 360° was applied to output images [20]. The summed values of the pixel intensity were plotted as a function of azimuthal angle, where the width (full width at half maximum: FWHM) is inversely proportional to the degree of orientation of the fibers.

#### 2.3.2. Tensile Test

Fiber substrates were punched out to dumbbell shape and a tensile test was performed using a uniaxial tensile machine (EZ Test EZ-SX, SHIMADZU, Kyoto, Japan). The electrospun fiber substrates (15 mm wide and 40 mm in length) were tested at a strain rate of 0.015 s^−1^ until fracture. The thickness of the fiber substrates was obtained between 10 and 20 μm. Elastic modulus (initial slope corresponding to <5% strain), ultimate strain, and fracture stress were calculated from a stress–strain curve.

#### 2.3.3. Crystallinity

The thermal properties were analyzed using the differential scanning calorimetry (DSC) (TA 2920, TA Instruments Co., New Castle, DE, USA) at a heating rate of 5 °C/min for PLLA and 1 °C/min for PCL fibers, respectively. The DSC was calibrated with Indium before experiments.

For the measurement of degree of crystallinity (𝑥_c_) prior to DSC analysis, the extra heat absorbed by the crystallites formed during heating had to be subtracted from the total endothermic heat flow due to the melting of the whole crystallites. Briefly the endothermic heat flow *ΔH*_difference_ of the initially existing crystallites can be calculated as *ΔH*_difference_ = *ΔH*_m_ − *ΔH*_c_, where *ΔH*_m_ is the endothermic melting enthalpy. *ΔH*_c_ is the exothermic ordering/crystallization enthalpy during heating process. The 𝑥_c_ was thus calculated as *ΔH*_difference_/*ΔH*° with *ΔH*° = 93 J/g for PLLA and *ΔH*° = 139.5 J/g for PCL, which is the melting enthalpy of 100% crystalline PLLA and PCL [21,22].

### 2.4. Cell Culture

Human breast adenocarcinoma cell line, MDA-MB-231 (ATCC, HTB-26) and MCF-7 (ATCC, HTB-22) were cultured in high glucose Dulbecco’s modified Eagle’s medium (DMEM) (Nacalai Tesque, Kyoto, Japan) supplemented with 10% (*v*/*v*) FBS, 100 unit/mL penicillin (Nacalai Tesque), and 100 μg/mL streptomycin (Nacalai Tesque), grown at 37 °C under 5% CO_2_ atmosphere and 95% relative humidity at 37 °C. Cells were grown to 70–80% confluence at normal culture condition before being seeded onto the fiber substrates.

### 2.5. Immunofluorescence Staining

For collagen gel and collagen-coated substrates, MDA-MB-231 and MCF-7 cells were seeded at the density of 1.0 × 10^4^ cells/cm^2^ on 96-well plates and cultured for a period of 24 h and 72 h. On the other hand, both cancer cells were seeded at the density of 1.0 × 10^4^ cells/cm^2^ on chamber slide and cultured for a period of 24 h, 48 h and 144 h for PLLA and PCL substrates prepared in the Section 2.2.2 and Section 2.2.3 Both cells were seeded on the spin coated flat substrates (designated as F-), random fibers (designated as R-) and aligned fibers (designated as A-) substrates.

Cells were fixed with 4% paraformaldehyde for 15 min at room temperature. Then cells were washed with phosphate buffer saline (PBS, Nacalai Tesque) and permeabilized with 0.1% Triton-X for 6 min. The fixed cells were washed twice with PBS and blocked with 2% bovine serum albumin (BSA) in PBS for 60 min.

For visualization of cell–cell adhesion, immunofluorescence staining was chosen. The cultured cells were treated with E-cadherin primary antibody (Takara Bio) over night at 4 °C and dyed with secondary antibody conjugated with Alexa Fluor 555 (Life Technologies) for 30 min at room temperature. Both antibodies were dissolved in 20 mM Tris buffer saline (TBS) supplemented 1% BSA and 10 mM CaCl_2_. The cell cytoskeleton and nuclei were stained by Alexa Fluor 488 phalloidin (Life Technologies) and Hoechst 33342 (Life Technologies) for 20 min. All stained samples were imaged by using a fluorescent microscope (EVOS FL Auto, Life Technologies).

Cellular morphologies (nuclear elongation factor, roundness, nuclear/cytoplasm) were quantified by following the contour of each cell manually (*n* = 32). Nuclear elongation factor, roundness and ratio of nuclear/cytoplasm were calculated as major axis/minor axis of nucleus, 4(area)/(π(major-axis)^2^) of cytoplasm, and area of nuclear/area of cytoplasm, respectively. By definition roundness is equal to 1 for a completely round cell.

### 2.6. Cell Proliferation

For collagen gel and collagen-coated substrates, MDA-MB-231 and MCF-7 cells were seeded at the density of 1.0 × 10^4^ cells/cm^2^ onto 96-well plates and cultured for a period of 24, 48 and 96 h in an atmosphere of 5% CO_2_ and 95% relative humidity at 37 °C. For PLLA and PCL substrates, 5 × 10^3^ cells/cm^2^ seeded on chamber slide and incubated for 12, 24, 72 and 144 h in an atmosphere of 5% CO_2_ and 95% relative humidity at 37 °C. At each time point, WST-8 assay (Dojindo, Tokyo, Japan) was assessed. Briefly, 10% WST-8/DMEM solution was added to the chamber slide and incubated for 1 h. Then the solution was transferred to a 96-well plate. The WST-8 colorimetric test was measuring the activity of intracellular dehydrogenase activity, which is proportional to living cells. The optical density was read on a Multiskan FC (Thermo Fisher Scientific, Tokyo, Japan) at 450 nm for absorbance and at 650 nm for subtract background absorbance.

### 2.7. Drug Sensitivity

MDA-MB-231 and MCF-7 cells were seeded at the density of 2.0 × 10^4^ cells/cm^2^ on collagen-coated and gel substrates and incubated for 24 h in an atmosphere of 5% CO_2_ and 95% relative humidity at 37 °C. Then subsequent drug treatments were performed. For PLLA and PCL substrates, both cells were seeded at the density of 5 × 10^3^ cells/cm^2^ on chamber slides and incubated for 72 h in an atmosphere of 5% CO_2_ and 95% relative humidity at 37 °C and subsequent drug treatments were performed to evaluate drug resistance.

Three different anti-cancer drugs (cisplatin, vorinostat and elrotinib) were diluted with each complete culture medium (DMEM) and added to each well. The concentrations used for this work were 0–100 µM for cisplatin, 0–5 µM for vorinostat and 0–20 µM for elrotinib.

After incubation for 72 h with each drug in an atmosphere of 5% CO_2_ and 95% relative humidity at 37 °C, the cell viability was assessed by WST-8 assay (Dojindo) according to manufacturer’s instructions. The optical density was read on a Multiskan FC (Thermo Fisher Scientific) at 450 nm for the absorbance and at 650 nm for the subtract background absorbance. The IC_50_ values at 72 h were estimated from the dose-response curves.

### 2.8. Statistics

All data presented are expressed as mean and standard deviations (±SD). Statistical analysis was performed using Student’s *t*-test and one-way analysis of variance with Dunnet’s post-hoc testing using Excel 2013 with Statcel4 v.2.0 Software (OMS Publishing Co., Tokyo, Japan), and significance was considered at a probability of *p* < 0.05.

## 3. Results

### 3.1. Effect of Substrate with Different Stiffness on Cellular Morphology

**Figure 1:**
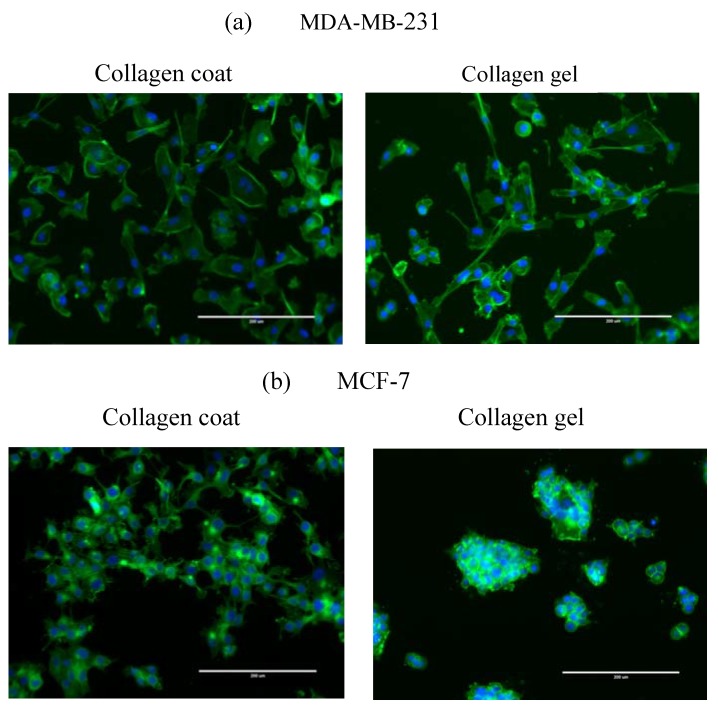
Morphological comparison of different type of breast cancer cell lines cultured on collagen- coated and collagen gel substrates for 3 days. (**a**) MDA-MB-231 and (**b**) MCF-7. Immunofluorescence of breast cancer cells were imaged with Hoechst 33342 (blue), phalloidin (green) Scale bar: 200 µm.

Figure 1 shows cellular morphology of MDA-MB-231 and MCF-7 breast cancer cells cultured on a stiffer collagen-coated and softer collagen gel substrates at day 3. For MDA-MB-231 cells incubated on both substrates, the cells are elongated along an arbitrary direction without the multicellular aggregate (colonization).

**Figure 2:**
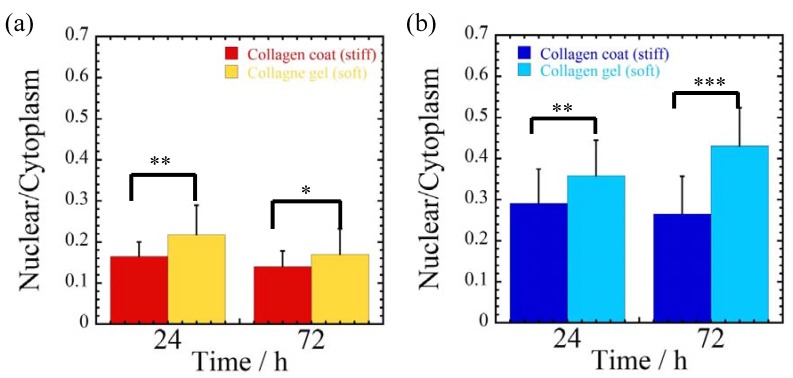
Quantification of cellular spreading parameters of (nuclear/cytoplasm) for (**a**) MDA-MB-231 and (**b**) MCF-7 cells cultured on collagen-coated and collagen gel substrates at different time intervals (24 and 72 h). *, ** and *** indicate *p* <0.05, 0.01 and 0.001, respectively.

In contrast, for MCF-7 cells incubated on collagen gel substrate, the cells are more aggregated than collagen-coated substrate. The corresponding cellular spreading parameters of nuclear/cytoplasm are summarized in Figure 2.

The ratio of nuclear area to cytoplasm area represents how much stress is transmitted to nucleus. MDA-MB-231 cells exhibit lower value of the ratios (nucleus/cytoplasm area ratio) for both substrates in comparison with MCF-7 cells, indicating the prominent cell stretching and more non-circular shape of the cellular morphology. For both cells, the spreading parameter (nucleus/cytoplasm area ratio) becomes greater for cells cultured on softer collagen gel substrate than stiffer collagen-coated substrate. This indicates that the cell stretching is restricted and the more circular morphology of the cells is overwhelming. In addition, both cells were more spread accompanied by lower values of spreading parameter with incubation time from 24 to 72 h, except MCF-7 cells cultured on collagen gel substrate. The response against collagen gel substrate for incubation of MCF-7 cells does not exactly follow the same trend of the feature of MDA-MB-231 cells.

For MCF-7 cells incubated on softer substrate (collagen gel), the difference in the spreading parameter at 72 h is more prominent (almost 1.6-fold higher as compared with stiffer substrate (collagen-coated)) in comparison with MDA-MB-231 cells. This is presumably due to the colonization, which leads to an enhanced cell–cell contact via E-cadherin, following the less traction forces (contractility) generation and transmitted stresses to nucleus occurring [23].

### 3.2. Effect of Substrates with Different Stiffness on Cellular Proliferation

**Figure 3:**
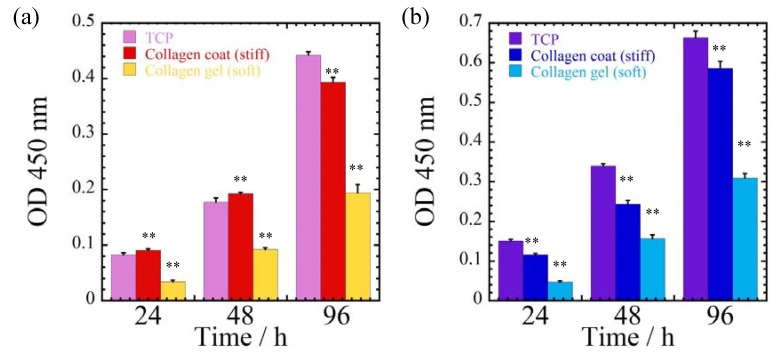
Optical densities (OD) of (**a**) MDA-MB-231and (**b**) MCF-7 cells cultured on different substrates (polystyrene tissue culture plates (TCP), collagen-coated, and collagen gel) at different time intervals (24, 48, and 96 h). ** indicates *p* <0.01 in comparison with TCP at each time.

Figure 3 shows the optical densities of MDA-MB-231 and MCF-7 cells cultured on different substrates (polystyrene tissue culture plates (TCP), collagen-coated, collagen gel) at different time intervals (24, 48 and 96 h). For all culture times, it is intriguing to observe that the collagen gel substrates have significant differences in cellular proliferation as compared to TCP and collagen-coated substrates for both MDA-MB-231 and MCF-7 cells, due to the smaller traction force generation.

### 3.3. Effect of Substrates with Different Stiffness on Drug Sensitivity

The IC_50_ values of three different types of anti-cancer drugs (cisplatin, vorinostat and elrotinib) are calculated from sigmoidal curves shown in Supplementary Figure S1. With increasing concentration up to 100 μM for cisplatin and 5 μM for vorinostat, a significant vitality reduction is observed as compared to control (Figure S1a,b,d,e). For the administration of elrotinib to MDA-MB-231 cells incubated on stiffer collagen-coated substrate, the cell viability is preserved (ca. 100% of cells survived) up to intermediate concentration of 5 μM for WST-8 test. Their viability is maintained at 70% for concentration up to 20 μM. Interestingly, the administration of elrotinib to the same cells on softer collagen gel substrate has high toxicity (damage) beyond concentration of 5.0 μM for 60% cell viability in the period of 24 h (Figure S1c).

By contrast, the mortality for the administration of elrotinib does not show such a concentration-dependent manner as for MCF-7 cells, indicating less toxic than cisplatin and vorinostat at comparable doses. The results show the addition of even as much as 20 μM of elrotinib in the cell culture did not kill the tested MCF-7 cells due to the drug resistant to elrotinib than MDA-MB-231 cells (Figure S1f). Elrotinib acts as an inhibitor of epidermal growth factor receptor (EGFR), which works effectively for lung cancer cells. However, many breast cancer cells are resistant to elrotinib such as BT-20, BT-549, MDA-MB-231, MDA-MB-468, and MCF-7 [24].

**Figure 4:**
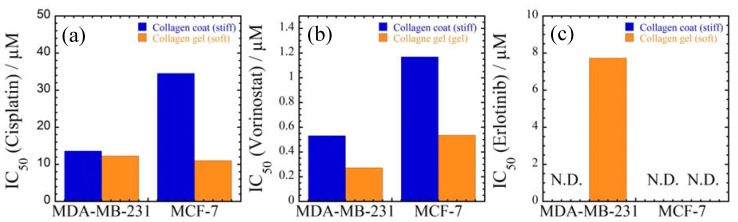
Summary of half maximal inhibitory concentration IC_50_ of MDA-MB-231 and MCF-7 cells cultured on collagen coated and collagen gel substrates after 72 h of incubation with (**a**) cisplatin; (**b**) vorinostat; and (**c**) elrotinib. N.D.: data are not detected.

The estimated IC_50_ values are summarized in Figure 4. The effect of the substrate stiffness between collagen gel and collagen-coated on the toxic feature is clearly observed. Although such difference in the MDA-MB-231 cells treated with cisplatin is not more evident (Figure 4a), the IC_50_ values for both cancer cells cultured on softer collagen gel substrate exhibits lower in toxicity as compared to that of cells cultured on stiffer collagen-coated substrate. The unelongated cellular morphologies lead to a significant vitality reduction.

### 3.4. Poly(l-lactic acid) PLLA and Poly(ε-caprolactone) Electrospun Nanofibers

**Figure 5:**
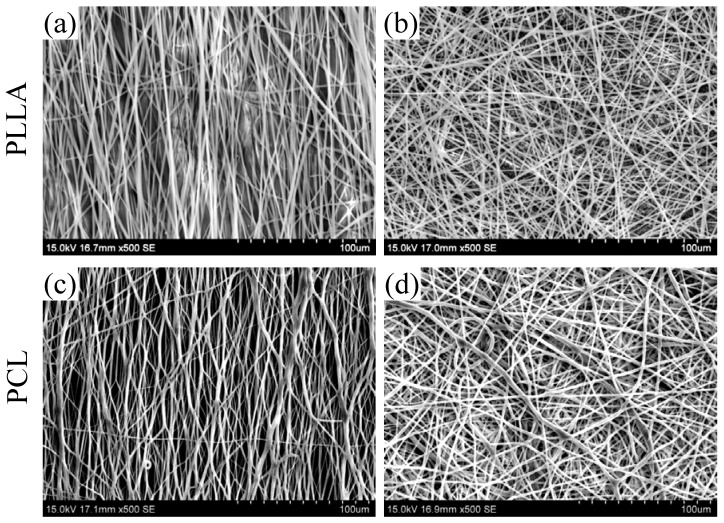
Field emission scanning electron microscope (FE-SEM) images showing electrospun fiber substrates: (**a**) Aligned PLLA (A-PLLA); (**b**) Random PLLA (R-PLLA); (**c**) Aligned PCL (A-PCL); and (**d**) Random PCL (R-PCL).

The morphology of obtained random and aligned electrospun polymeric fibers are shown in Figure 5. All electrospun fibers have uniform, bead-free, and smooth surface morphology with average fiber diameters of ~1.5 μm.

The characteristics for these fiber substrates derived from FE-SEM micrographs, tensile test and DSC measurement are summarized in Supplementary Table S1. The average fiber diameters of A-PLLA and R-PLLA fibers are around 1.5 μm, whereas R-PCL fiber mat exhibits larger values as compared to that of A-PCL. The decrease in the fiber diameter can be attributed to an elongation of the fiber during the collecting process. This effect is more prominent in the electrospun PCL fibers.

The reciprocal value of full width at half maximum (FWHM) is proportional to the degree of orientation of the fibers. Owing to the branched structure of electrospun A-PCL fiber (Figure 5c), the A-PCL fiber shows a lower degree of orientation compared to A-PLLA fiber.

Elastic modulus, fracture stress, and ultimate strain were assessed by a stress–strain curve of tensile test. The aligned fibers have significantly stronger tensile properties compared with random electrospun fibers. The random orientation of the fibers improves the ultimate strain at break in each mat created by electrospinning. The degree of the crystallization of PLLA and PCL for aligned and random fibers is calculated to be 53.7% for A-PLLA, 42.8% for R-PLLA, 32.4% for A-PCL, and 31.4% for R-PCL, respectively. The fiber orientation during collecting process in aligned fiber might accelerate the crystallization in the molecular level in electrospun PLLA fiber.

### 3.5. Effect of Fiber Topography and Stiffness on Cellular Morphology

**Figure 6:**
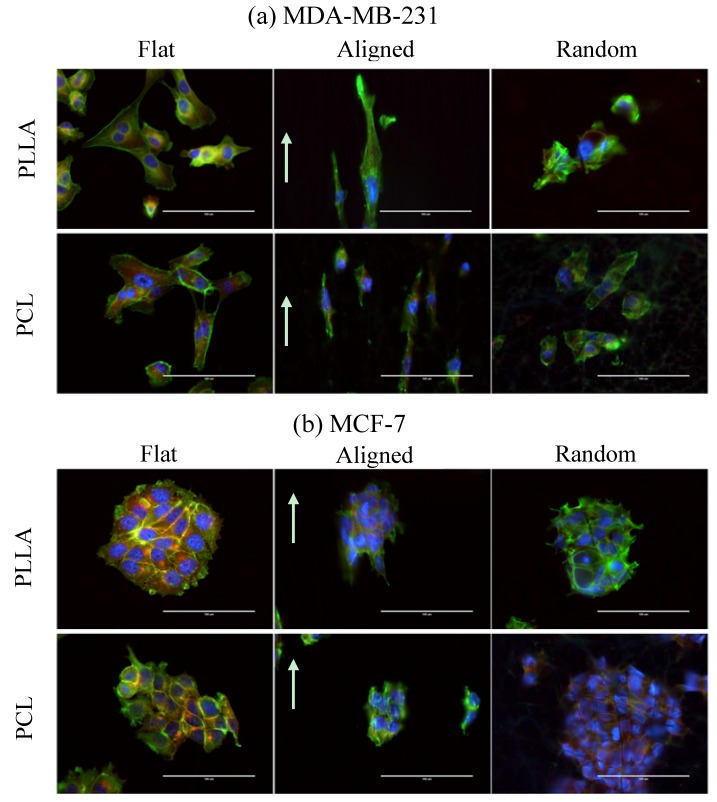
Morphological comparison of different type of breast cancer cell lines cultured on different substrates for 3 days. (**a**) MDA-MB-231 and (**b**) MCF-7. Immunofluorescence of breast cancer cells was imaged with Hoechst 33342 (blue), phalloidin (green), and E-cadherin antibody (red). Scale bar: 100 µm. Arrows indicate the aligned fiber direction of the substrate.

**Figure 7:**
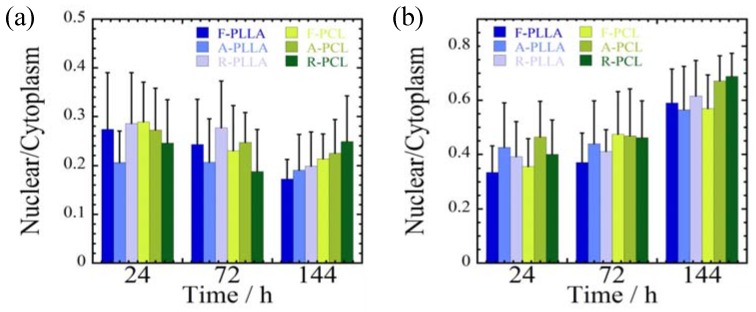
Quantification of cellular spreading parameters of (nuclear/cytoplasm) for (**a**) MDA-MB-231 and (**b**) MCF-7 cells cultured on F-, A-, and R-PLLA and/or F-, A-, and R-PCL substrates at different time intervals (24, 72 and 72 h).

Figure 6 shows cellular morphology of MDA-MB-231 and MCF-7 breast cancer cells cultured on different substrates at day 3. The corresponding cellular spreading parameters (nucleus/cytoplasm area ratio) are shown in Figure 7. For MCF-7 cells incubated on all substrates, E-cadherin (red fluorescence) was detectable and localized at the intercellular boundaries (borders), indicating the multicellular aggregate (colonization) of the cells (Figure 6b). On the other hand, such aggregation of MDA-MB-231 cells is not observed (Figure 6a), but the cells are elongated and arranged to fiber direction when the cells are incubated on the aligned fibers substrates. The aligned fiber substrates produce less elongation and alignment of the MCF-7 cells along fiber orientation directions than MDA-MB-231 cells (Figure 6b). This phenomenon was similar to the results reported by Saha et al [25].

Both cells cultured on random fiber substrates (R-PLLA and R-PCL) produce fewer spread morphologies compared to the other substrates at 144 h (Figure 7). For both MDA-MB-231 and MCF-7 cells cultured on R-PCL, a larger value of the spreading parameter (nucleus/cytoplasm area ratio) is observed at 144 h (Figure 7). This indicates that cell stretching is restricted with R-PCL substrate. Furthermore, MDA-MB-231 cells can be more deformed than MCF-7 cells due to an enhanced metastatic potential.

### 3.6. Effect of Aligned Substrates with Stiffness on Cellular Proliferation

**Figure 8:**
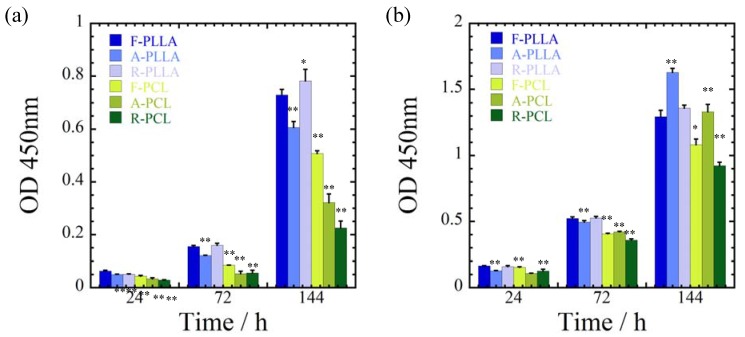
Optical densities of (**a**) MDA-MB-231and (**b**) MCF-7 cells cultured on different (F-, A- and R-PLLA, and F-, A- and R-PCL) substrates at different time intervals (12, 24, 72, and 144 h). * and ** indicate *p* < 0.05 and 0.01, respectively in comparison with F-PLLA substrate at each time.

Figure 8 shows the optical densities of MDA-MB-231 and MCF-7 cells cultured on different substrates at different time intervals (12, 24 and 144 h). For all culture times, it is noted that the combination of both surface topographies and stiffness of the substrate have significant differences in cellular proliferation, although there is no clear cell dependency between both cancer cells. For instance, the PLLA substrates have significant cellular proliferation as compared to PCL substrates for both cancer cells, while the surface topography of the aligned fibers substrates induces the enhancement of MCF-7 cells proliferation in comparison with MDA-MB-231 cells.

### 3.7. Effect of Aligned Substrates with Stiffness on Drug Sensitivity

**Figure 9:**
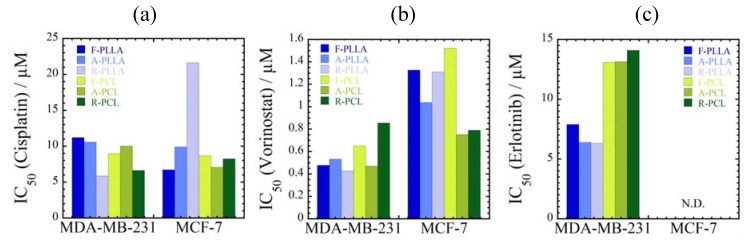
Summary of IC_50_ of MDA-MB-231 and MCF-7 cells cultured on different (F-, A- and R-PLLA, and F-, A- and R-PCL) substrates treated with (**a**) cisplatin; (**b**) vorinostat; and (**c**) elrotinib.

The IC_50_ values of three different types of anti-cancer drugs are estimated from the sigmoidal curve shown in Supplementary Figure S2. The administration of anti-cancer drugs exhibits growth inhibition in a concentration-dependent manner as observed in Figure S1. Even large amount of added elrotinib (up to 20 μM) did not show toxicity, and more than 90% cells survived (Figure S2f). The calculated IC_50_ are presented in Figure 9.

Both cancer cells show different IC_50_ values when they were cultured on different substrates. Especially, MCF-7 cells cultured on R-PLLA exhibit higher resistance against cisplatin compared with other substrates (2 to 3 times higher in IC_50_ values). The IC_50_ values of MDA-MB-231 cells are less affected by each substrate. These results are consistent with the levels of cell viability as described in Section 3.3. As mentioned above, MCF-7 cells are more responsive to elrotinib than MDA-MB-231 cells [23] (Figure 9c). Note that erlotinib works more effectively with MDA-MB-231 cells incubated on PLLA substrates in comparison with PCL substrates. Taken together, cellular drug sensitivity seems to be affected by the combination of both stiffness and topography of substrates.

## 4. Discussion

Extensive studies have been made in a mechanotransduction via surface topography and stiffness on the substrates, in which cells respond to applied forces and exert forces in the substrate (ECM) [25,26,27]. Such forces can change cell morphology and cytoskeletal structure due to traction forces (contractility) generation, which influence cell response and cell fate. Apart from this, nuclear factor κB (NF-κB) activation is associated with the spreading parameter (nucleus/cytoplasm area ratio) [27]. NF-κB is known to be involved in inflammatory and tumor development, including cellular proliferation, apoptosis and drug sensitivity [8,26,27,28,29,30]. Additionally, several groups reported that NF-κB was regulated by actomyosin contractility [28,31,32]. Some of the mentioned studies may be helpful to consider that the proliferation is regulated by NF-κB activation via actomyosin contractility.

**Figure 10:**
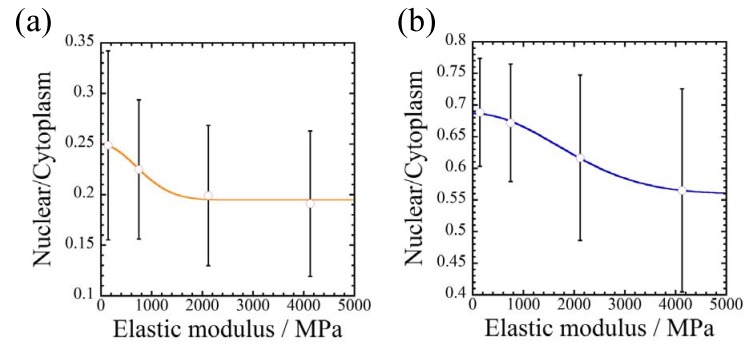
Effect of substrate stiffness on cell spreading, i.e., the ratio (nucleus/cytoplasm) for (**a**) MDA-MB-231 and (**b**) MCF-7 cells incubated on F-, A-, and R-PLLA and/or F-, A-, and R-PCL substrates at 72 h.

As mentioned above, each of the substrate conditions in this study prompt us to examine the proliferation and drug responsiveness of both breast cancer cells cultured on different substrates with different culture times. To elucidate the effect of substrate stiffness on cell spreading, the spreading parameters (nucleus/cytoplasm area ratio) are plotted as a function of elastic moduli of each fiber substrate (Figure 10). Owing to the difference of the stiffness between PLLA and PCL substrates (15-fold), cellular elongation is caused by the stiffness and surface topographies used to achieve high efficacy in this experiment. These results support the important roles of the substrate stiffness as well as local stiffness of materials obtained from atomic force microscopy (AFM) measurement [33,34,35].

**Figure 11:**
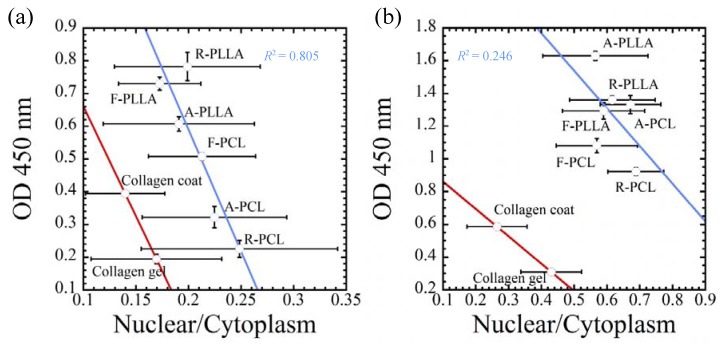
Relationship between cellular proliferation and spreading parameters (nucleus/cytoplasm area ratio) for (**a**) MDA-MB-231 and (**b**) MCF-7. Optical densities were employed at the time point of 96 h and spreading parameters of (nuclear/cytoplasm) were employed at the time point of 72 h for both cancer cells cultured on collagen-coated and collagen gel substrates. Optical densities and spreading parameters of (nuclear/cytoplasm) were employed at the time point of 144 h for both cancer cells cultured on F-, A-, and R-PLLA and/or F-, A-, and R-PCL substrates.

The traction forces via surface topography and stiffness of the substrates can control the proliferation of both cells [27,36]. In this regard, the optical densities are plotted as the spreading parameter (nucleus/cytoplasm area ratio) on each substrate at the day 6 point (144 h) for PLLA and PCL substrates and at the day 4 point (96 h) for collagen-coated and gel substrates (Figure 11). It is important to note that incubation with stiff substrates produces significant cell growth activity by the traction force generation, supporting the level of morphological parameter (nucleus/cytoplasm are ratio). Both cells are producing significant proliferation associated with the cellular spreading factor (nucleus/cytoplasm area ratio). It is important to note that cells feeling greater traction forces produce larger cell growth activity. These results correspond to the report of Sero et al. that NF-κB activation is associated with the parameter nucleus/cytoplasm area ratio [28].

**Figure 12:**
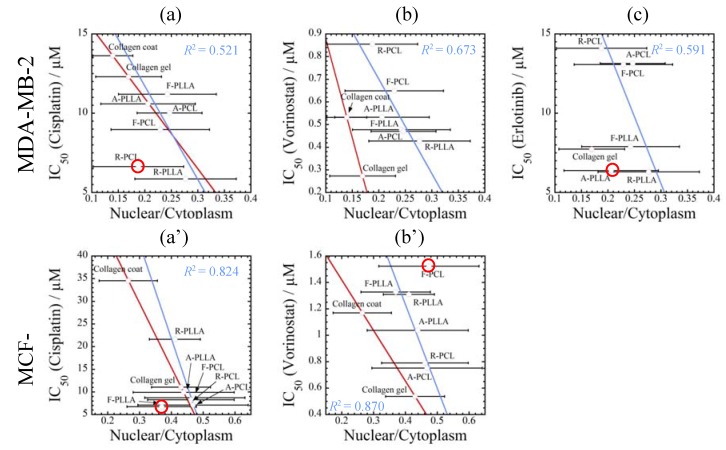
Relationship between IC_50_ (Figure 4 and Figure 9) of three different anti-cancer drugs (**a,a’**) cisplatin; (**b,b’**) vorinostat; and (**c,c’**) elrotinib and spreading parameters of (nuclear/cytoplasm) at 72 h for (**a**–**c**) MDA-MB-231 and (**a’**,**b’**) MCF-7cells incubated on different (collagen, PLLA, and PCL) substrates. Red circles indicate the deviation from linear regression.

The morphological change induced by underlying substrates also might influence the drug responsiveness of the cells. Thus, the IC_50_ values are plotted as a function of the spreading factor (nucleus/cytoplasm area ratio) at the day 3 point to elucidate the effect of substrates on drug sensitivity comprehensively (Figure 12). Blue solid lines indicate the IC_50_ values of cells cultured on PLLA and PCL substrates (Figure 9) and red solid lines indicate the IC_50_ values of cells cultured on collagen-coated and gel substrates (Figure 4). For both cancer cells and all anti-cancer drugs, the IC_50_ values are increased with decreasing in the parameter (nucleus/cytoplasm area ratio), supporting intimate correlation between morphological parameter and drug responsiveness. Especially, a significant drug sensitivity for cisplatin of MDA-MB-231 cells reflects the reduction in the parameter (nucleus/cytoplasm area ratio) (Figure 12a,d), regardless of the different culture conditions (i.e., PLLA and PCL substrates, collagen substrates, and culture time).

Overall, cellular proliferation and drug resistance are enhanced by decreasing the parameter (nucleus/cytoplasm area ratio). Thus, the parameter (nucleus/cytoplasm area ratio) reflects the metastatic spread of cancer cells (proliferation), supported especially well by preventing them from cell death (IC_50_ values).

## 5. Conclusions

The combination of both surface topographies (fiber alignments) and different levels of stiffness of the polymeric substrates (PLLA and PCL) and/or collagen substrates (coating and gel) has been employed to evaluate the effect of the cellular morphology on proliferation and drug responsiveness of two different types of breast cancer cells.

The spreading parameter (nucleus/cytoplasm area ratio) was chosen to elucidate the effect of the substrate characteristics on cellular morphology. The incubation with stiff substrates (PLLA and collagen-coated) produces significant cell growth activity by the traction force generation, supporting the level of morphological parameter (nucleus/cytoplasm area ratio). For both cancer cells and all anti-cancer drugs (cisplatin, vorinostat and elrotinib), the IC_50_ values were increased by decreasing in the parameter (nucleus/cytoplasm area ratio). The intimate correlation between spreading parameter and drug sensitivity was demonstrated regardless of the different culture conditions (i.e., PLLA and PCL substrates, collagen substrates, and culture time).

The present study has examined the observation of cellular morphology induced by physical, mechanical properties and topographies of a variety of substrates.

Biological phenomena, including cancer progression and metastatic potential, are quite complex. Although the difficulty of the strategies lies in interpretation of the local microenvironment situation of ECM for malignant tumor cells, the morphological spreading parameter (nucleus/cytoplasm area ratio) induced by the anthropogenic substrates is expected when one applies the parameter for the evaluation of cancer cell malignancy.

## Supplementary Materials

Supplementary materials related to this article can be found, in the online version, at http://www.mdpi.com/2079-4983/8/2/18/s1, Figures S1 and S2, Table S1.
