# Supplementary material for: Cellular Morphology-Mediated Proliferation and Drug Sensitivity of Breast Cancer Cells
Source: J Funct Biomater. 2017 Jun 6;8(2):18. doi: 10.3390/jfb8020018 (PMC5491999; doi:10.3390/jfb8020018)
Supplement: Supplementary file 1 [file jfb-08-00018-s001.pdf]

Cellular morphology-mediated proliferation and drug sensitivity of breast cancer cells

Ryota Domura,[†] Rie Sasaki,[†] Yuma Ishikawa, [†] Masami Okamoto,[†, *]

[†]Advanced Polymeric Nanostructured Materials Engineering, Graduate School of Engineering, Toyota Technological Institute, 2-12-1 Hisakata, Tempaku, Nagoya 468 8511, Japan

Supplementary data: Table S1, Figures S1, S2.

Table S1. Morphological parameters, tensile properties and degree of crystallinity of polymeric fiber-based substrates.

Substrates	Fiber diameter (μm)	FWHM (°)	Elastic modulus (GPa)	Fracture stress (MPa)	Ultimate strain (%)	Crystallinity (%)
F-PLLA	-	-	-			-
A-PLLA	1.48 ± 0.31	44.33	4.1	112	20	53.7
R-PLLA	1.54 ± 0.29	-	2.1	91	78.	42.8
F-PCL	-	-	-			-
A-PCL	1.37 ± 0.49	55.22	0.75	67	48	32.4
R-PCL	1.86 ± 0.62	-	0.15	25	209	31.4

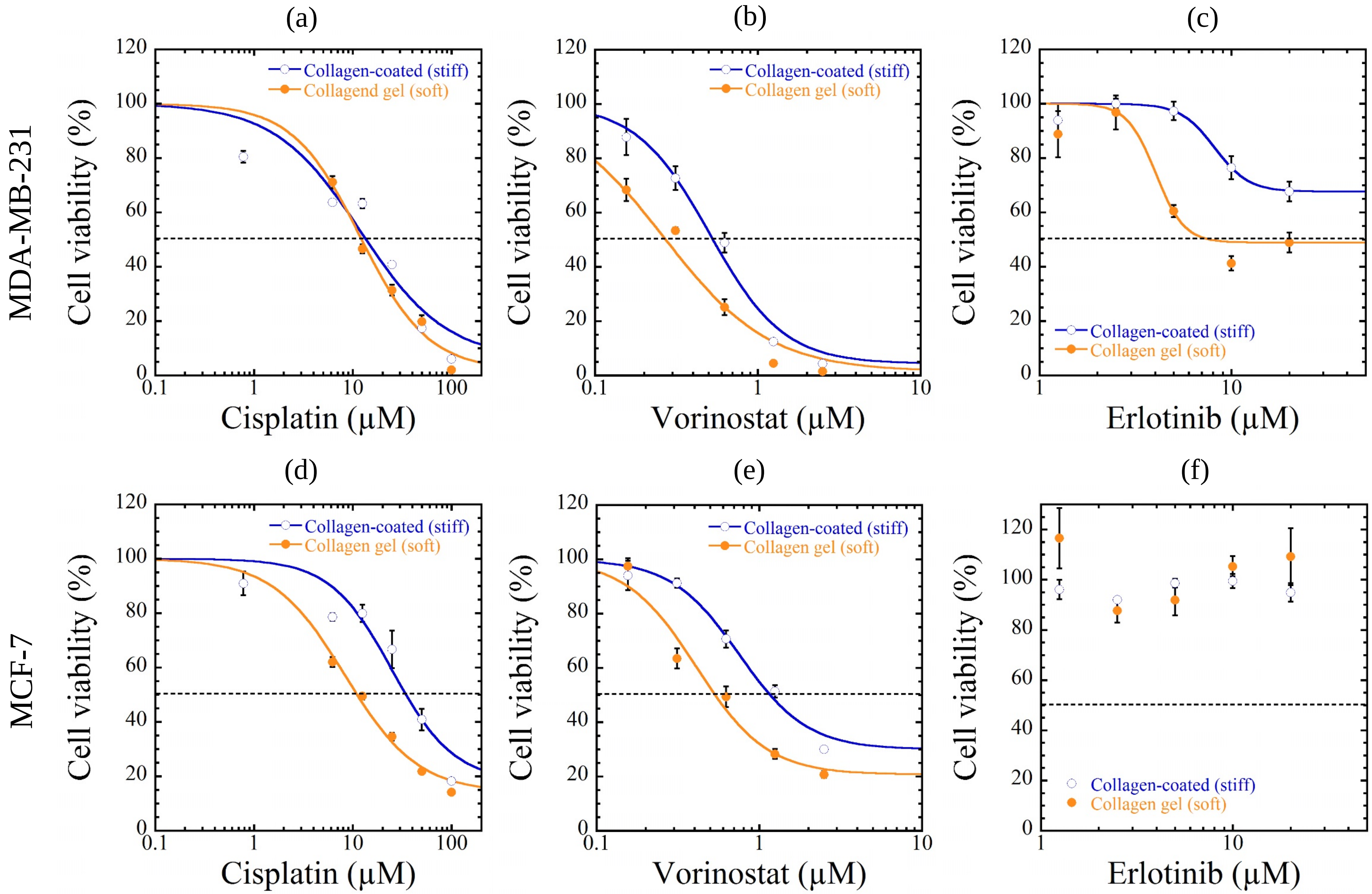

Figure S1. Cell viability as measured by WST-8 assay using ((a)–(c)) MDA-MB-231 and ((d)–(f)) MCF-7cells incubated on collagen-coated and gel substrates after 72 h of incubation with (a, d) cisplatin, (b, e) vorinostat, and (c, f) eltotinib of different concentrations.

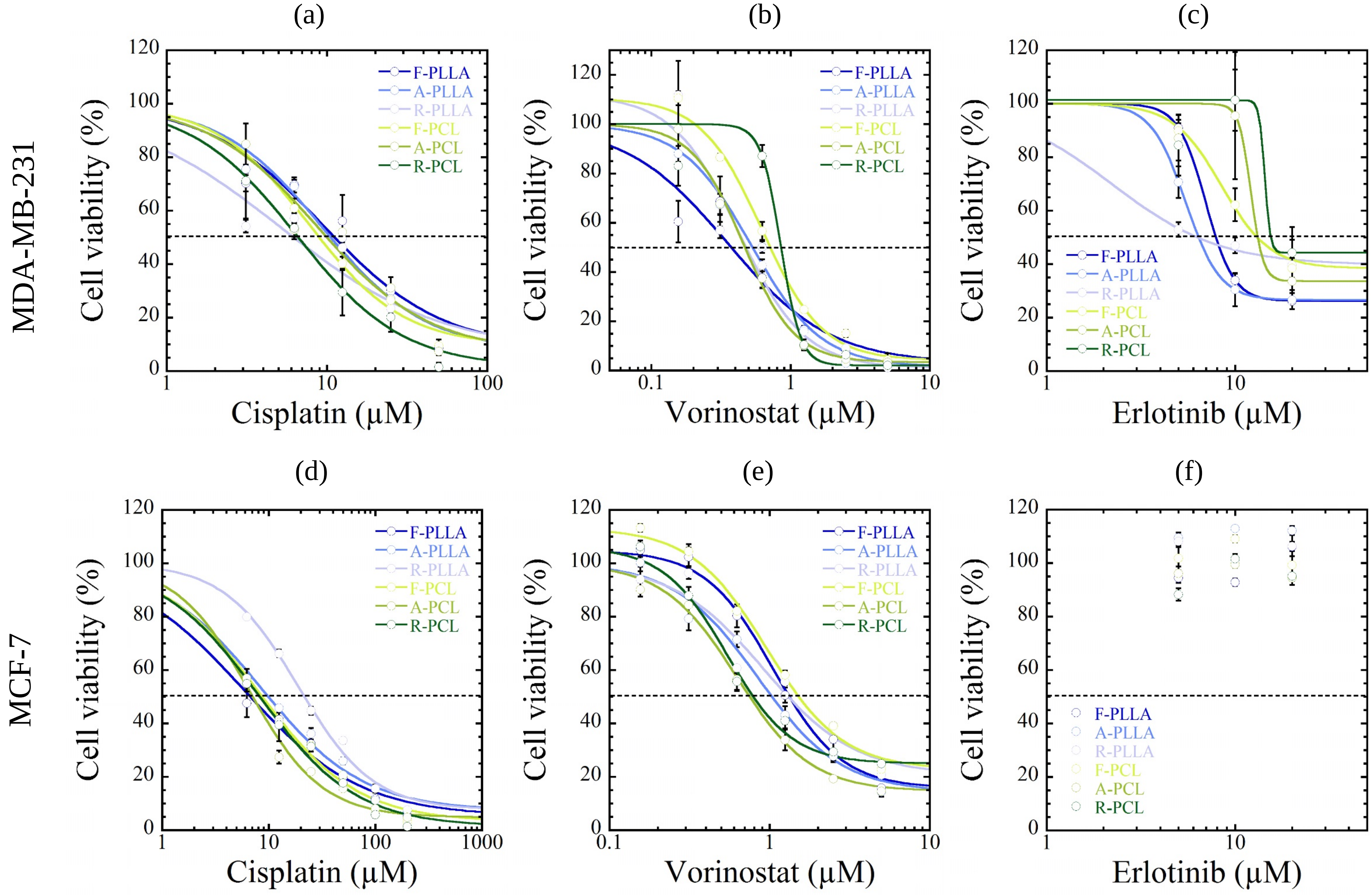

Figure S2. Cell viability as measured by WST-8 assay using ((a)–(c)) MDA-MB-231 and ((d)–(f)) MCF-7 cells incubated on F-, A-, and R-PLLA and/or F-, A-, and R-PCL substrates after 72 h of incubation with (a, d) cisplatin, (b, e) vorinostat, and (c, f) eltotinib of different concentrations.
